# Effect of Different Temperatures on Herbicide Efficacy for the Management of the Invasive Weed *Solanum rostratum* Dunal (Family: Solanaceae)

**DOI:** 10.3390/plants14040574

**Published:** 2025-02-13

**Authors:** Jackline Abu-Nassar, Maor Matzrafi

**Affiliations:** Department of Plant Pathology and Weed Research, Newe Ya’ar Research Center, Agricultural Research Organization—Volcani Institute, Ramat Yishay 30095, Israel

**Keywords:** fluroxypyr, metribuzin, oxyfluorfen, tembotrione, weed control

## Abstract

*Solanum rostratum* Dunal, an invasive weed first recorded in Israel in the 1950s, undergoes multiple germination waves from early spring to late summer. Recently, its distribution has significantly expanded, with new populations reported throughout the country. This study assessed the efficacy of various herbicides for controlling *S. rostratum* populations under two distinct temperature regimes, focusing on temperature-dependent variations in herbicide performance. The results demonstrated that fluroxypyr and tembotrione consistently achieved high levels of control across all temperature conditions. Conversely, oxyfluorfen exhibited low performance under elevated temperatures and showed greater population-specific variability, while metribuzin proved more effective at higher temperatures across all *S. rostratum* populations. These findings emphasize the pivotal role of post-application temperature in influencing herbicide efficacy and underscore the importance of a precise application timing to optimize the control outcomes. Temperature-optimized herbicide strategies could play a critical role in limiting the spread and establishment of *S. rostratum* in agricultural systems, contributing to a sustainable and effective weed management.

## 1. Introduction

*Solanum rostratum* (common name, buffalobur) belongs to the Solanaceae family and is a native species of the Mexican highlands [1] that has invaded several countries worldwide, including Canada, China, Russia, and Australia [2]. *S. rostratum* is a summer annual, C3, self-compatible weed species with prickles on both leaves and stems. It is usually found in open, disturbed habitats, such as roadsides, fallow fields, and field margins [2]. In the native range of the US, this weed was reported as an agricultural pest in Oklahoma [3], Nebraska, and Wyoming [4]. Field experiments conducted in cotton fields in Oklahoma showed that crop plant heights were decreased at greater densities of *S. rostratum* [3]. In the same study, yield reduction was also observed, with a damage of 480 kg ha^−1^ at a weed density of 64 plants per 10 m row.

In Israel, *S. rostratum* was first documented in the Jezreel Valley in 1953 [5]. Since then, several field populations were located in the Jordan Valley, the Golan Heights, the Hulla Valley, and on the Mediterranean Sea coastline [6]. In Israel, *S. rostratum* is considered a major pest of *Citrullus lanatus*, *Allium cepa*, *Zea mays*, and *Solanum lycopersicum*. It can appear in several germination flashes, starting from spring and continuing throughout the summer; thus, high or low temperatures can alter the effectiveness of chemical weed control.

Herbicide effectiveness is highly dependent on environmental conditions, which influence herbicide absorption, penetration, translocation, and detoxification. Changes in environmental factors including temperature, precipitation, radiation level, relative humidity, dew, wind, and soil moisture may affect herbicide efficacy both directly and indirectly [7,8,9,10]. Thus, the environmental conditions must be optimal during herbicide application to ensure effective herbicide absorption, penetration, and translocation. In addition, non-optimal conditions in the time interval after herbicide application may also alter the plant response to the herbicide treatment. For instance, in most cases, post-application high temperatures decrease herbicide efficiency, particularly for herbicides that are easily detoxified by the plant metabolism, such as glyphosate [11,12], metolachlor [13], glufosinate [14], mesotrione [15], pinoxaden [16], and rimsulfuron [17]. Only in the case of paraquat, did post-application low temperatures restrict translocation and lead to reduced herbicide efficacy [18,19]. Recent studies underscore the importance of tailored herbicide applications to effectively manage this invasive species while minimizing adverse effects on the surrounding vegetation and reducing the overall herbicide usage [6,20]. This study explores the efficacy of various herbicides for the control of *S. rostratum*, focusing on seven distinct field populations collected across Israel. Specifically, we examine the temperature-dependent performance of four herbicides—oxyfluorfen, metribuzin, fluroxypyr, and tembotrione—depending on post-application temperature.

## 2. Results

**Figure 1:**
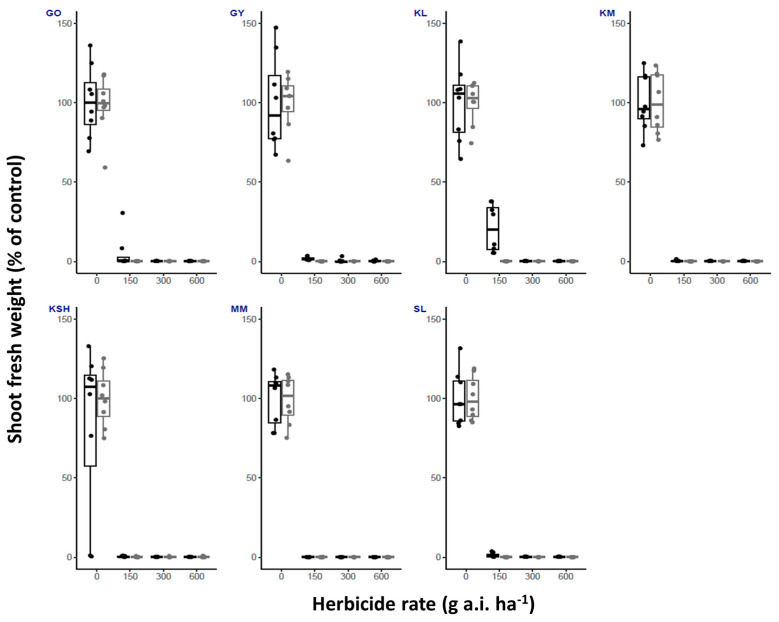
Shoot fresh weight (% of control) of *S. rostratum* plants from seven populations (GO, GY, KL, KM, KSH, MM, SL) treated with three rates of fluroxypyr (1/2X, X and 2X, 300 g a.i. ha^−1^ is the recommended labeled field rate as specified in Table 4). Plants were grown under high and low temperature regimes (black and gray bars, respectively). *n* = 8.

**Figure 2:**
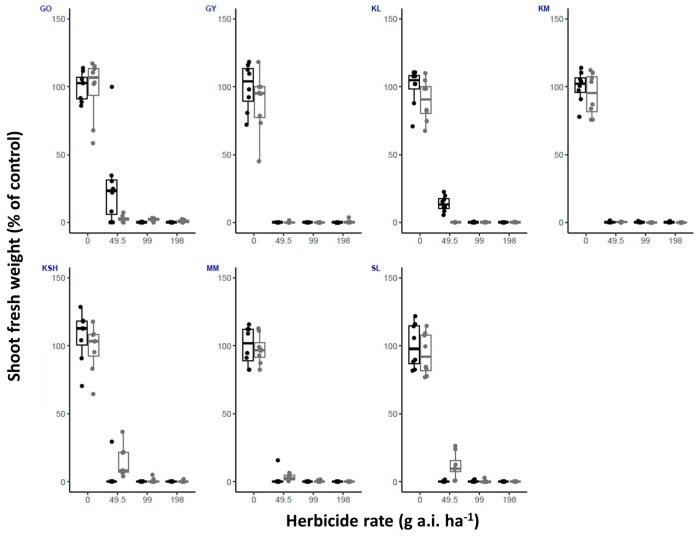
Shoot fresh weight (% of control) of *S. rostratum* plants from seven populations (GO, GY, KL, KM, KSH, MM, SL) treated with three rates of tembotrione (1/2X, X and 2X, 99 g a.i. ha^−1^ is the recommended labeled field rate as specified in Table 4). Plants were grown under high and low temperature regimes (black and gray bars, respectively). *n* = 8.

This study confirmed the importance of temperature for optimized herbicide use and timing of application for the proper control of *S. rostratum*. We show that fluroxypyr (Figure 1) and tembotrione (Figure 2) provided consistent results across both high and low temperature conditions. Both herbicides showed good control of plants from all seven populations examined.

For fluroxypyr, no survivors were recorded at any of the used herbicide rates, apart from 25% of survivors at half of the recommended labeled field rate shown for the GO population at high temperatures (Table 1).

The same trend was recorded for tembotrione, with high efficacy for both temperature regimes (Figure 2). However, at half of the recommended labeled field rate, survival was up to 50% for the KL and SL populations, and no clear correlation with temperature was observed.

**Figure 3:**
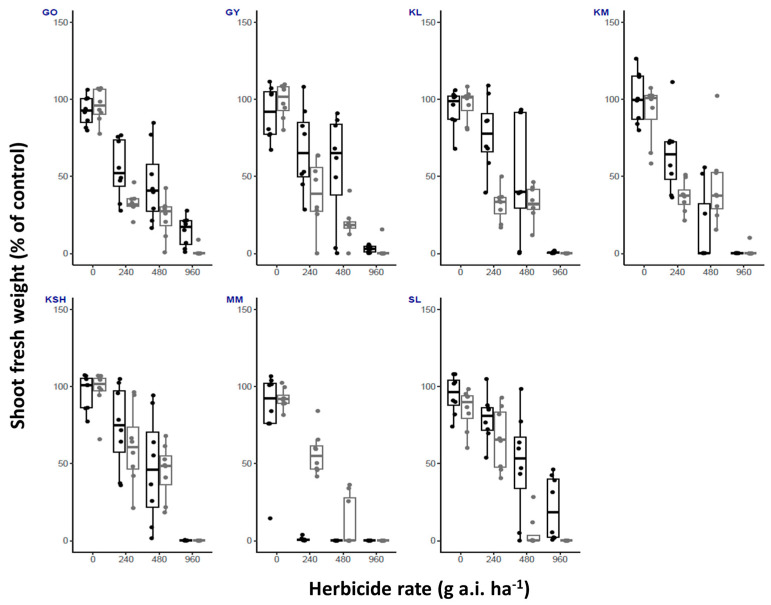
Shoot fresh weight (% of control) of *S. rostratum* plants from seven populations (GO, GY, KL, KM, KSH, MM, SL) treated with three rates of oxyfluorfen (1/2X, X and 2X, 480 g a.i. ha^−1^ is the recommended labeled field rate as specified in Table 4). Plants were grown under high and low temperature regimes (black and gray bars, respectively). *n* = 8.

**Figure 4:**
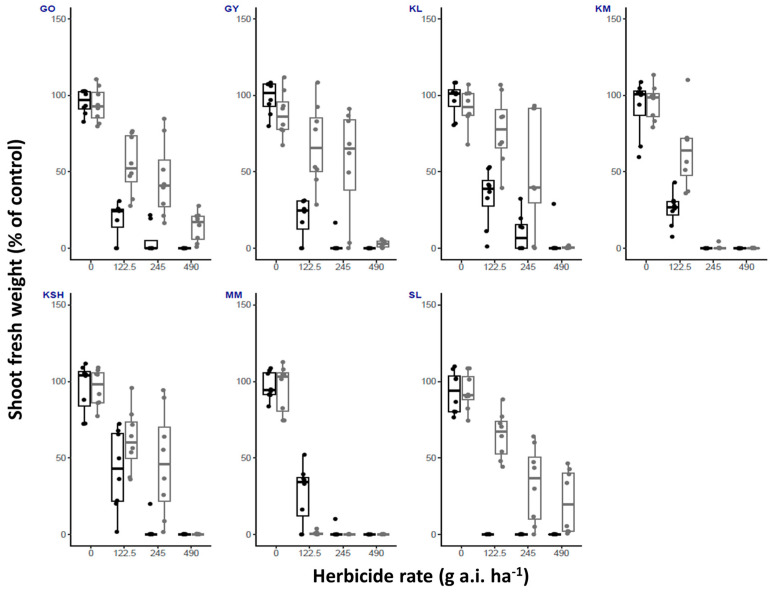
Shoot fresh weight (% of control) of *S. rostratum* plants from seven populations (GO, GY, KL, KM, KSH, MM, SL) treated with three rates of metribuzin (1/2X, X and 2X, 245 g a.i. ha^−1^ is the recommended labeled field rate as specified in Table 4). Plants were grown under high and low temperature regimes (black and gray bars, respectively). *n* = 8.

Oxyfluorfen and metribuzin demonstrated temperature-related herbicide efficacy, as shown in Figure 3 and Figure 4, respectively, and Table 1. Oxyfluorfen showed higher control rates when the plants were grown under low temperatures; this was shown especially for the populations GO, GY, and SL. The populations KM and MM showed the opposite trend, with high control levels when the plants were grown under high temperatures (Figure 3). For the KM and MM populations, higher survivor percentages were recorded under low temperature at the recommended labeled field rate (100% and 50%, respectively; Table 1). At the high rate of 960 g a.i. ha^−1^, high survival percentages were recorded for the GO (87.5%) and SL (50%) populations with the high temperature treatment.

Metribuzin performed better when the plants were grown under high temperature, achieving a reduction in shoot fresh weight that was observed for all populations (Figure 4).

An analysis of the fresh shoot weight of plants treated with the recommended field-labeled rate of metribuzin revealed a statistically significant reduction in shoot fresh weight across all populations except for KM and MM (Table 2). For these two populations, herbicide efficacy was exceptionally high at the recommended rate, with no surviving plants recorded following treatment (Table 1).

## 3. Discussion

Herbicide performance is significantly influenced by environmental factors, which affect key processes like absorption, penetration, translocation, and detoxification. Ensuring favorable conditions during and after herbicide application is essential, as adverse conditions can impair these processes and diminish effectiveness [21].

In our study, temperature emerged as a critical factor influencing the efficacy of both oxyfluorfen and metribuzin, demonstrating population-specific and temperature-dependent responses in *S. rostratum*. For oxyfluorfen, lower temperatures increased herbicide efficacy in certain populations (e.g., GO, GY, and SL), whereas elevated temperatures enhanced its effectiveness in others (e.g., KM and MM). Apart from the temperature-dependent herbicide response, the variability observed in our study suggests differential oxyfluorfen sensitivity among *S. rostratum* populations, potentially due to genetic or physiological differences. For instance, at the high rate of 960 g a.i. ha^−1^, the survival percentages were notably high for the GO (87.5%) and SL (50%) populations under elevated temperature conditions. Previous studies highlighted that herbicide permeability differences, driven by elevated levels of cuticular waxes in mature compared to young leaves of *S. rostratum*, can significantly affect herbicide efficacy [21]. This reduced efficacy under elevated temperatures may be further exacerbated by the extensive deposition of intra-cuticular waxes in mature leaves, which can act as a barrier to herbicide absorption and contribute to herbicide resistance [20,22].

Metribuzin, on the other hand, exhibited a consistent pattern of higher efficacy at elevated temperatures across all populations. This aligns with studies on *S. lycopersicum* (tomato), where increased temperatures intensified metribuzin-induced injury [23,24]. However, contrasting results were reported for *Amaranthus albus* populations, where temperature had no effect on metribuzin sensitivity [17]. This may suggest that these differences are related specifically to Solanaceae species.

Reduced sensitivity to pesticides under altered environmental conditions was previously documented [25]. In populations of the weed *Avena sterilis* ssb. *ludoviciana*, reduced efficacy of clodinafop propargyl and mesosulfuron-methyl + iodosulfuron-methyl was detected under drought stress conditions [26]. For *A. fatua*, reduced efficacy of fenoxaprop was detected under drought stress conditions [27]. *Chenopodium album*, *Conyza canadensis*, and *C. bonariensis* also exhibit altered herbicide translocation under high temperatures, which leads to reduced herbicide efficacy [11,12]. The environmental conditions may also affect herbicide metabolism through various detoxification pathways. Reduced sensitivity of *B. hybridum* to treatment with pinoxaden (an acetyl-CoA carboxylase inhibitor) under elevated temperatures was found to be correlated with herbicide metabolism [16]. The physiological and genetic mechanisms involved in these responses remain largely unknown. It is important to mention that the effect may vary among herbicide-susceptible and resistant populations. In populations of *Lolium* spp., for example, tested for their response to diclofop-methyl under increasing temperatures, the effect of elevated temperature was more pronounced in herbicide-resistant plants than in susceptible ones [16]. Previous findings have been reported for *Hordeum glaucum*, where the efficacy of paraquat varied between resistant (R) and susceptible (S) biotypes depending on temperature, highlighting the interaction between environmental conditions and population traits [18].

Weeds are organisms capable of quickly adapting to new, challenging environments [28,29]. They typically have a generalist strategy of survival, where genotypes respond to environmental cues by expressing advantageous phenotypes that allow them to thrive [30,31]. It is predicted that phenotypic plasticity (the ability of a plant genotype to express multiple phenotypes in response of abiotic and biotic cues) evolves more often when populations experience spatial and temporal environmental heterogeneity [32,33], such as that experienced by weeds in agroecosystems. Weed populations are, therefore, more likely to acclimate to novel extreme weather events and selective pressures caused by climate change then other plant species and crops [34].

Given the significant role of temperature and population variability in herbicide efficacy, farmers must consider these factors when planning herbicide applications. For example, metribuzin was more effective under high temperatures, while oxyfluorfen performed better under low temperatures. Based on these findings, farmers could apply oxyfluorfen earlier in the season when temperatures are lower and metribuzin later in the season when temperatures are higher. Aligning herbicide use with environmental conditions could improve the control of *S. rostratum*. Optimizing herbicide use under local environmental conditions can improve control outcomes, reduce the risk of resistance development, and minimize environmental impacts. This is particularly important for invasive weeds like *S. rostratum*, whose adaptability poses significant challenges to agricultural systems under future projected climate change scenarios. Future research should explore the physiological and genetic mechanisms underlying temperature-dependent herbicide responses and population variability. Understanding these interactions will enable the development of more targeted and effective weed management strategies, ensuring sustainable agricultural practices in the face of climate change and evolving weed populations.

## 4. Materials and Methods

### 4.1. Plant Material

Seeds of *S. rostratum* were collected in 2023 from seven different locations, as specified in Table 3. The berries were crushed, and the seeds were cleaned and dried at room temperature and then stored at 4 °C until use. In order to break seed dormancy, we used a two-stage protocol, as specified in Abu-Nassar and Matzrafi [6]. In the first stage, the seeds were immersed in a sulfuric acid solution at 95–97% concentration for 10 min. After the treatment, the seeds were rinsed thoroughly with distilled water and dried at room temperature. In the second stage, the seeds were soaked in 2.4 mM gibberellic acid (GA3) at room temperature and in dark conditions for 24 h. After the dormancy-breaking treatments, the seeds were sown in 300 mL pots filled with commercial potting medium (Tuff, Marom Golan, Israel), including the Osmocote^®^ slow-release fertilizer, at the New-Ya’ar Research Center. To test the response to the herbicides under different temperatures, the plants were grown under two temperature regimes, i.e., at high temperatures (34.43 °C ± 5.77 during the day and 25.82 °C ± 1.72 at night) and at low temperatures (19.27 °C ± 5.27 during the day and 16.02 °C ± 2.79 at night). The plants were watered daily.

### 4.2. Herbicide Application

The experiments were conducted in a greenhouse at the Newe-Yaar Research Center. Plants from seven populations were sprayed when they reached the height of 4–5 cm. Plants from each population were treated with four different herbicides at three different rates, i.e., the recommended rate (specified in Table 4) and half and twice the recommended labeled field rate. The herbicides were applied using a chain-driven sprayer that delivered 300 L ha^−1^, equipped with a flat-fan 8001E nozzle (TeeJet^®^, Spraying Systems Co., Wheaton, IL, USA). One hour post herbicide application, the plants were moved back to the greenhouse. All experiments were arranged in a fully randomized factorial design, with eight replicates for each treatment. The shoot fresh weight and survival rate of each plant were recorded 21 days after treatment (DAT).

### 4.3. Statistical Analyses

The response of the populations to the herbicide treatments, measured by shoot fresh weight, was analyzed using Student’s *t*-test with the ggsummarystats R package [35]. A *p*-value ≤ 0.05 was considered to indicate statistically significant differences among the treatments. The *t*-test function was used for the analysis, and data visualization was carried out using the ggboxplot function from the same R package.

## Figures and Tables

**Table 1:** Percentage of survival for all treatment and temperature regimes.

	Herbicide/ Temperature	Fluroxypyr				Metribuzin				Oxyfluorfen				Tembotrione			
Pop ID	Rate (g a.i. ha^−1^)	0	150	300	600	0	122.5	245	490	0	240	480	960	0	49.5	99	198
GO	High	100	25	0	0	100	37.5	25	0	100	100	100	87.5	100	12.5	0	0
	Low	100	0	0	0	100	100	100	62.5	100	100	87.5	12.5	100	25	0	0
GY	High	100	0	0	0	100	75	12.5	0	100	100	75	25	100	0	0	0
	Low	100	0	0	0	100	100	25	12.5	100	87.5	75	12.5	100	0	0	0
KL	High	100	0	0	0	100	100	50	0	100	100	50	0	100	50	0	0
	Low	100	0	0	0	100	100	87.5	0	100	100	100	0	100	0	0	0
KM	High	100	0	0	0	100	100	0	0	100	100	50	0	100	0	0	0
	Low	100	0	0	0	100	100	0	0	100	100	100	12.5	100	0	0	0
KSH	High	100	0	0	0	100	100	12.5	0	100	100	75	0	100	0	0	0
	Low	100	0	0	0	100	100	75	0	100	100	100	0	100	25	0	0
MM	High	100	0	0	0	100	25	0	0	100	12.5	0	0	100	0	0	0
	Low	100	0	0	0	100	0	0	0	100	100	50	0	100	0	0	0
SL	High	100	0	0	0	100	0	0	0	100	100	87.5	50	100	0	0	0
	Low	100	0	0	0	100	100	62.5	50	100	100	25	0	100	50	0	0

**Table 2:** Shoot fresh weight (mean ± SE) of seven populations of *S. rostratum* treated with the recommended field rates of various herbicides, measured under two different temperature regimes (high and low). *n* = 8.

Pop ID	Herbicide/ Temperature	Fluroxypyr		Metribuzin		Oxyfluorfen		Tembotrione	
	Rate (g a.i. ha^−1^)	0	300	0	245	0	480	0	99
GO	High	100.69 ± 22.70 a	0.28 ± 0.00 b	95.61 ± 7.70 a	5.23 ± 9.53 c,d	92.62 ± 9.54 a	45.29 ± 24.82 b,c	100.42 ± 10.42 a	0.35 ± 0.20 c
	Low	98.34 ± 18.49 a	0.19 ± 0.00 b	93.80 ± 11.29 a	45.22 ± 24.79 b	96.13 ± 10.67 a	23.89 ± 12.93 d,e	98.48 ± 17.11 a	2.22 ± 0.00 c
GY	High	99.92 ± 29.46 a	0.52 ± 1.16 b	98.50 ± 10.67 a	2.18 ± 5.89 c,d	91.05 ± 9.54 a	55.50 ± 24.82 b,c	100.00 ± 13.88 a	0.327 ± 0.20 c
	Low	99.91 ± 17.96 a	0.11 ± 0.00 b	87.84 ± 14.81 a	55.62 ± 35.88 b	96.13 ± 10.67 a	18.78 ± 12.93 d	88.27 ± 11.50 a	0.45 ± 0.34 c
KL	High	100.03 ± 24.13 a	0.26 ± 0.02 c	97.43 ± 10.84 a	10.05 ± 12.04 d	93.81 ± 17.10 a,b	49.60 ± 35.81 c	96.39 ± 11.47 a	0.35 ± 34.80 d
	Low	100.00 ± 13.63 a	0.16 ± 0.01 c	91.89 ± 12.39 a,b	49.52 ± 38.76 c	96.81 ± 10.97 a	31.80 ± 11.30 c	89.95 ± 13.91 b	0.35 ± 0.01 d
KM	High	100.00 ± 17.77 a	0.30 ± 0.00 b	92.18 ± 18.50 a	0.07 ± 0.01 d	101.12 ± 12.70 a	16.86 ± 38.82 d	100.08 ± 16.27 a	0.44 ± 0.70 b
	Low	100.04 ± 18.61 a	0.09 ± 0.01 c	95.61 ± 11.56 a	0.76 ± 1.54 d	91.56 ± 10.26 a	37.07 ± 11.10 c	94.41 ± 22.52 a	0.15 ± 1.08 b
KSH	High	99.89 ± 73.50 a	0.27 ± 0.03 b	96.39 ± 11.47 a	2.68 ± 34.80 d	96.39 ± 16.74 a	46.99± 4.52 c	107.04 ± 22.15 a	0.28 ± 0.00 c
	Low	100.13 ± 17.61 a	0.25 ± 0.24 b	96.39 ± 11.47 a	46.99 ± 34.80 b,c	97.67 ± 13.63 a	45.14 ± 17.54 d	98.7 ± 15.16 a	1.00 ± 0.00 c
MM	High	100.17 ± 16.34 a	0.16 ± 0.01 b	97.01 ± 8.98 a	1.35 ± 3.57 c	83.02 ± 30.37 a	0.16 ± 0.01 c	100.41 ± 15.40 a	0.16 ± 0.00 b
	Low	99.26 ± 15.04 a	0.10 ± 0.06 b	95.40 ± 15.56 a	0.16 ± 0.00 c	92.28 ± 13.63 a	12.06 ± 17.54 c	97.07 ± 16.94 a	0.47 ± 1.78 b
SL	High	100.30 ± 17.13 a	0.34 ± 0.02 b	93.19 ± 13.63 a	0.13 ± 0.01 d	94.81 ± 12.54 a	49.42 ± 33.71 c	97.46 ± 10.51 a	0.475 ± 0.59 b
	Low	100.30 ± 17.13 a	0.34 ± 0.02 b	93.45 ± 12.11 a	32.75 ± 24.92 c	85.12 ± 13.43 a	5.19 ± 10.25 d,e	94.22 ± 15.32 a	0.36 ± 1.00 c

Different lowercase letters indicate statistically significant differences among treatments, as determined by a *t* test (α = 0.05); shoot fresh weight was recorded 21 days after herbicide application.

**Table 3:** Population ID and location across Israel. Description of the specific habitats and characteristics.

Pop ID	GPS Coordinates	*Specific Crop/Habitat
Kibbutz Ginegar (GO)	32.6543271802, 35.2488696828	watermelon
Moshav Givat Yoav (GY)	32.8012266548, 35.6977272346	watermelon
Kibbutz Lavi (KL)	32.7874152565, 35.4297385362	maize
Kfar Masaryk (KM)	32.8851355473, 35.109928859	tomato
Kibbutz Sha’alabim (KSH)	31.8722352816, 34.9683375942	corn
Kibbutz Ma’agan Michael (MM)	32.5472419936, 34.9184483341	Fallow
Kfar Sold (SL)	33.194514, 35.642185	Fallow

*Specific crop/habitat refers to the field where seeds were collected.

**Table 4:** List of herbicides used in this study and their recommended labeled field rates.

Common Name	Trade Name	MOA ^a^	Manufacturer	Rate (g a.i. ha^−1^)
Fluroxypyr	Tomahawk ^®^	PPO inhibitors	ADAMA-Agan	300
Metribuzin	Sencor^®^	PSII inhibitor	Bayer	245
Oxyfluorfen	Galigan^®^	PPO inhibitors	ADAMA-Agan	480
Tembotrione	Laudis^®^	HPPD	Bayer	99

^a^ MOA—Herbicide mode of action.

## Data Availability

Data will be provided upon request.
